# Life history of northern Gulf of Mexico Warsaw grouper *Hyporthodus nigritus* inferred from otolith radiocarbon analysis

**DOI:** 10.1371/journal.pone.0228254

**Published:** 2020-01-24

**Authors:** Beverly K. Barnett, Jeffrey P. Chanton, Robert Ahrens, Laura Thornton, William F. Patterson

**Affiliations:** 1 National Marine Fisheries Service, Southeast Fisheries Science Center, Panama City Laboratory, Panama City, Florida, United States of America; 2 University of Florida, Fisheries and Aquatic Sciences, Gainesville, Florida, United States of America; 3 Florida State University, Earth, Ocean and Atmospheric Science, Tallahassee, Florida, United States of America; 4 Riverside Technology, Inc. Fort Collins, Colorado, United States of America under contract to National Marine Fisheries Service, Southeast Fisheries Science Center, Panama City Laboratory, Panama City, Florida, United States of America; Australian Bureau of Agricultural and Resource Economics and Sciences, AUSTRALIA

## Abstract

Warsaw grouper, *Hyporthodus nigritus*, is a western Atlantic Ocean species typically found at depths between 55 and 525 m. It is listed as a species of concern by the U.S. National Marine Fisheries Service and as near threatened by the International Union for the Conservation of Nature. However, little information exists on the species’ life history in the northern Gulf of Mexico (nGOM) and its stock status in that region is currently unknown. Age of nGOM Warsaw grouper was investigated via opaque zone counts in otolith thin sections (max age = 61 y), and then the bomb ^14^C chronometer was employed to validate the accuracy of age estimates. Otolith cores (n = 14) were analyzed with accelerator mass spectrometry and resulting Δ^14^C values overlain on a loess regression computed for a regional coral and known-age red snapper Δ^14^C time series. Residual analysis between predicted Δ^14^C values from the loess regression versus Warsaw grouper otolith core Δ^14^C values indicated no significant difference in the two data series. Therefore, the accuracy of otolith-based aging was validated, which enabled growth and longevity estimates to be made for nGOM Warsaw grouper. Dissolved inorganic carbon (DIC) Δ^14^C values collected from the nGOM support the inference that juvenile Warsaw grouper occur in shelf waters (<200 m) since DIC Δ^14^C values in this depth range are enriched in ^14^C and similar to the Δ^14^C values from otolith cores. A Bayesian model was fit to fishery-dependent age composition data and produced von Bertalanffy growth function parameters of L_∞_ = 1,533 mm, k = 0.14 y^-1^, and t_0_ = 1.82 y. Fishing mortality also was estimated in the model, which resulted in a ratio of fishing to natural mortality of 5.1:1. Overall, study results indicate Warsaw grouper is a long-lived species that is estimated to have experienced significant overfishing in the nGOM, with the age of most landed fish being <10 y.

## Introduction

Fisheries for deepwater reef fishes exist on outer continental shelf or upper slope habitats around the globe, with many deepwater species considered to be vulnerable to exploitation due to longevity, slow growth, late maturity, and low natural mortality (M) [1−4]. In addition, some groups of reef fishes, such as several grouper species (Family: Serranidae), form spawning aggregations which makes them highly vulnerable to fishing [1, 5−6]. Many of these species are considered to be data-poor due to a lack of life-history data; thus, their stock status is often unknown. Several international workshops have been held to discuss ways to not only improve life history parameter estimates [7−8], but also to improve monitoring strategies for many of these data-poor, deepwater fishes [9].

Age is perhaps the most influential biological life-history parameter since it provides the basis for estimating growth and mortality rates [10]. Therefore, evaluation of the accuracy and precision of age estimates is critical to fisheries ecology and stock assessment [11−12]. Otoliths from deepwater reef fishes have opaque increments that are often difficult to interpret which can lead to age estimates that are biased low. In turn, this affects the reliability of age estimates, results in biased estimates of longevity (too low) and M (too high), and uncertain estimates of productivity. Therefore, the importance of accurate age estimates cannot be overstated since ages are not only fundamental to estimating life-history parameters, such as growth, mortality, and longevity, but also have important implications for estimating sustainable harvest rates for exploited species.

Traditional methods used to verify or validate annual opaque zone formation in otoliths are problematic for deepwater species given year-round samples are often not available for marginal increment analysis, and chemically marking otoliths is impractical given the deep waters of outer shelf and upper slope habitats adults occupy. However, bomb radiocarbon (^14^C) produced from atmospheric testing of nuclear weapons during the 1950s – 1960s [13] has been successfully used to validate the accuracy of age estimates for several deepwater species [14–16]. This method relies on the rapid increase in ^14^C that occurred in the atmosphere due to weapons testing that was subsequently absorbed via air-sea diffusion into the mixed layer of oceanic waters [13]. Thereafter, this ^14^C signal was incorporated into the aragonite skeletons of hermatypic corals [17], which provides a reference series to which otolith Δ^14^C can be compared to validate the accuracy of fish age estimation [18].

Warsaw grouper is a deepwater species encountered on reefs located in 55 to 525 m of water throughout the temperate to tropical western Atlantic Ocean, including the Gulf of Mexico (GOM) and Caribbean Sea [19]. It is a protogynous hermaphrodite (i.e., changing sex from female to male) [20], and reaches a maximum size of approximately 2.3 m total length and a body mass of 200 kg [19]. The species was classified as endangered with a high risk of extinction by the American Fisheries Society [21]. Thereafter, the National Marine Fisheries Service (NMFS) listed Warsaw grouper as a species of concern in 2004, and the International Union for Conservation of Nature (IUCN) listed it as near threatened in 2018 [22].Unfortunately, little information exists on its life history, including in U.S. waters of the northern GOM (nGOM) where its stock status is currently unknown [22].

The goal of this study was to investigate the life history and population ecology of Warsaw grouper in the nGOM via analysis of archived otolith samples. Specific objectives included: 1) producing age estimates of Warsaw grouper from sectioned otolith samples; 2) validating the accuracy of age estimation via application of the bomb radiocarbon chronometer; 3) drawing inference about likely juvenile habitat through comparison of otolith core Δ^14^C values with regional coral-otolith Δ^14^C time series and dissolved inorganic carbon (DIC) Δ^14^C values collected from the nGOM; and, 4) estimating nGOM Warsaw grouper growth and mortality rates. Study results have important implications for Warsaw grouper management and conservation.

## Materials and methods

### Ethics statement

NMFS Animal Care and Use Policy (04–112) is currently limited to research on free-living marine mammals, seabirds, and sea turtles and does not cover research on captive or wild fish. However, fish samples used in this study were collected and handled in strict accordance within the guidelines of the *U*.*S*. *Government Principles for the Utilization and Care of Vertebrate Animals Used in Testing*, *Research and Training (**https*:*//olaw*.*nih*.*gov/sites/default/files/PHSPolicyLabAnimals*.*pdf**)* and the *American Fisheries Society Guidelines for the Use of Fishes in Research (**https*:*//fisheries*.*org/docs/policy_useoffishes*.*pdf**; Chapter V)*.

### Data collection

**Fig 1:**
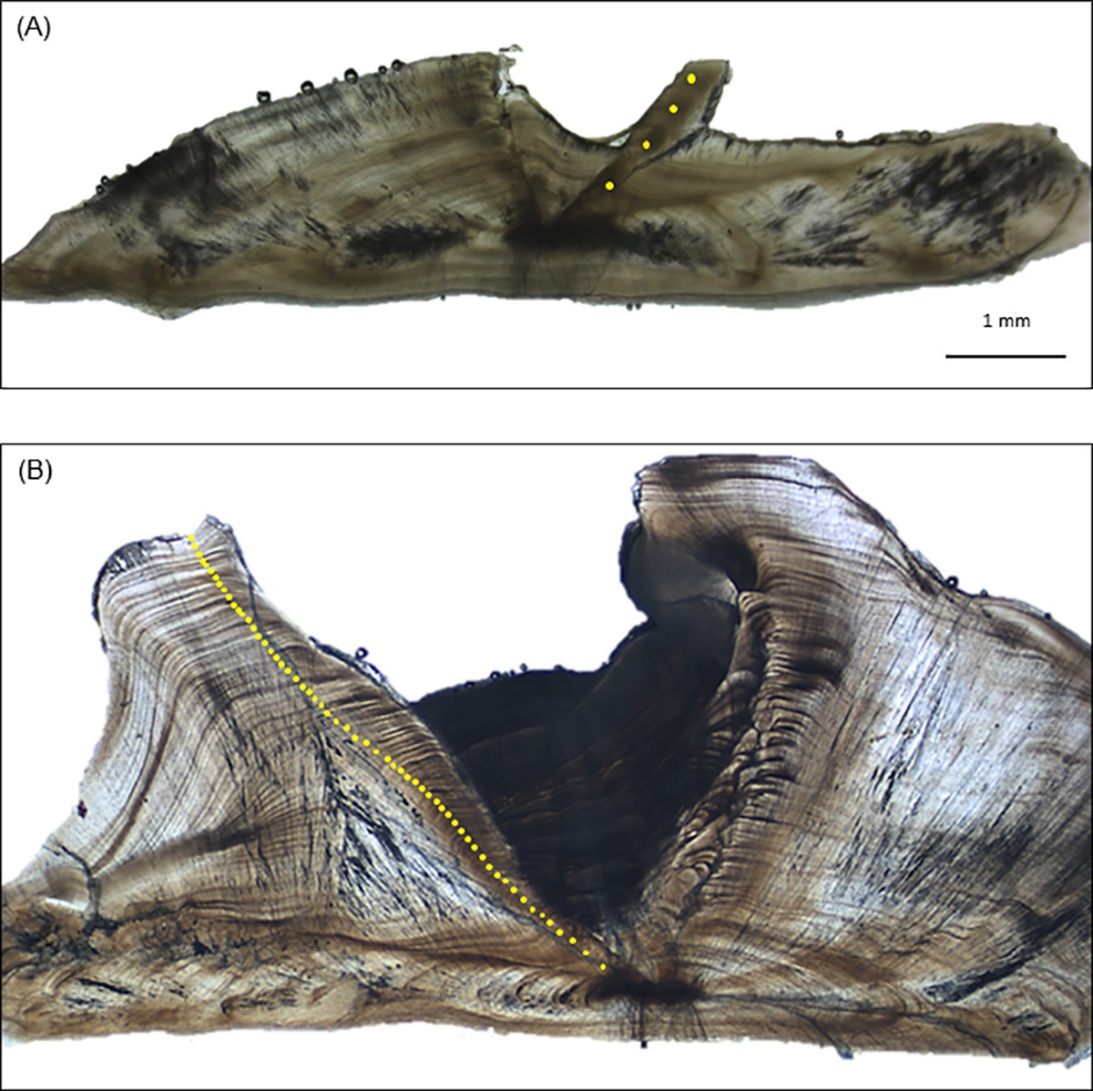
Images of Warsaw grouper otolith sections. Images of Warsaw grouper sagittal otolith sections viewed with transmitted light. Panel A is from a 974 mm TL fish estimated to be 4 years old. Panel B is from a 1,954 mm TL fish estimated to be 59 years old and for which the core of the right otolith was extracted and analyzed for Δ^14^C (sample WRG-0675). Scale bar shown in Panel A is the same for Panel B.

Warsaw grouper sagittal otolith samples were collected from 1980 to 2017 during fishery-dependent and fishery-independent sampling programs conducted in the nGOM from Texas to the Florida Keys. In the eastern nGOM, samples were collected between 24°N– 30°N, 81°W– 89°W. In the western nGOM, samples were collected between 26°N– 29°N, 89°W– 97°W. Fishery-dependent sampling was conducted by NMFS port agents, Florida Wildlife Research Institute port agents, and NMFS at-sea observers assigned to bottom longline and hook-and-line commercial fishing vessels. Most (95%) of the samples were landed in the commercial and recreational fisheries, with an additional 1% of the samples being sampled from fishing tournaments. Fishery-dependent samples were collected under the regulatory authority of the Code of Federal Regulations (CFR), 50 CFR Part 622.2 and 622.5. Fishery-independent samples, which comprised approximately 4% of the samples, were provided from bottom longline, trap, trawl and hook-and-line surveys conducted by NMFS Pascagoula, NMFS Panama City, and Louisiana Department of Wildlife and Fisheries. Fishery-independent samples were collected under Scientific Research Permits issued from the NMFS Southeast Regional Office in accordance with the definitions and guidance of 50 CFR 600.10 and 600.745. All fishery-dependent and fishery-independent samples were collected from public waters in the nGOM. Fish total length (TL) was measured and otoliths were extracted and stored dry in paper coin envelopes and archived at the NMFS Panama City Laboratory. Otoliths were subsequently weighed, and a transverse section of 0.5 mm thickness was made through the core region (Fig 1). All otoliths were read with transmitted light using a Leica S8 APO microscope at 15x to 20x magnification to count opaque zones, which constituted the age estimate for a given sample. Due to the difficulty in assigning edge types, ages were estimated based on counts of opaque zones alone. All otoliths were read by one reader without knowledge of length, otolith mass, or date of capture. A subsample of otolith sections was randomly selected and read by a second reader without knowledge of fish size or the first reader’s opaque zone counts. Average percent error (APE), percent reader agreement, and coefficient of variation (CV) were calculated between readers as measures of aging precision [23].

A subsample (n = 14) of otolith samples was selected for Δ^14^C analysis. Samples were selected in an attempt to validate age estimates for young (decline period) and old (rise period) fish using bomb radiocarbon Δ^14^C analysis. To validate age estimates for longevity, samples were selected based on otoliths that had the heaviest otolith mass, as this is often the metric that is more indicative of an older fish compared to using fish length alone. Once samples were selected, left otoliths were sectioned for age estimates. Right otoliths were embedded in an epoxy resin, which was allowed to cure for 48 hours prior to further preparation for radiocarbon analysis. A transverse section of 1.5 mm thickness containing the otolith core was made and the section then mounted to a glass slide with Loctite glue. The slide was affixed to the milling stage with paraffin wax, the otolith core was then identified, and extraction of the core utilized the computer-automated capabilities of a New Wave Research® (ESI–NWR Division; Fremont CA 94538 USA) micromill instrument following the method of Barnett and Patterson [24]. A 0.5 mm diameter Brasseler® (Savannah, GA 31419 USA) bit was used to remove the otolith core as a single piece. Once extracted, core material was weighed and stored dry in acid-leached glass vials.

### Data analyses

Otolith samples were analyzed for Δ^14^C and δ^13^C with accelerator mass spectrometry (AMS) at the National Ocean Sciences Accelerator Mass Spectrometry (NOSAMS) facility at the Woods Hole Oceanographic Institution. Processing and analysis of otolith samples for Δ^14^C proceeded at NOSAMS following standard methods (additional information can be found online: www.whoi.edu/nosams/radiocarbon-data-calculations). The delta value δ^13^C (‰) is calculated as the ratio of ^13^C/^12^C relative to a standard (Vienna Pee Dee Belemnite). The delta value for radiocarbon reported as Δ^14^C (‰) represents the activity of a sample relative to a standard [25] that has been corrected for age and δ^13^C-corrected for fractionation.

A Caribbean and GOM coral Δ^14^C reference series was combined with known-age GOM red snapper *Lutjanus campechanus* otolith samples [26], which extend the post-peak decline time series to 2015, to create a coral-otolith reference time series to compare Warsaw grouper otolith core Δ^14^C values. Coral reference sample sites included Belize [27], the Florida Keys [28], Vera Cruz, Mexico [29], the Flower Garden Banks off Texas [29], and Puerto Rico [30]. The combined coral-otolith Δ^14^C time series was fit with a loess regression (degree = 2, α = 0.20) in the program R [31], and measured values of Warsaw grouper otolith Δ^14^C were then added to the coral-otolith plot at their respective birth years to test the accuracy of Warsaw grouper age estimates. Confidence intervals were Bonferroni-corrected and estimated around the loess regression [32]. Assumptions for normality and homogeneity of variance were tested with Shapiro-Wilk and Levene tests, respectively. Significance levels were set at α = 0.05.

Age estimates for cored Warsaw grouper otolith samples analyzed for Δ^14^C were investigated for aging bias by purposely shifting the age estimates by ±1 to 3 years. Adding positive age bias shifted age estimates to older ages, while negative age bias shifted age estimates to younger ages. Original age estimates were represented as age shift of 0. Thereafter, the sum of squared residuals (SSR) for each age shift was calculated by subtracting observed birth year (based on opaque zone counts) from predicted birth year for cored otolith samples [32].

**Fig 2:**
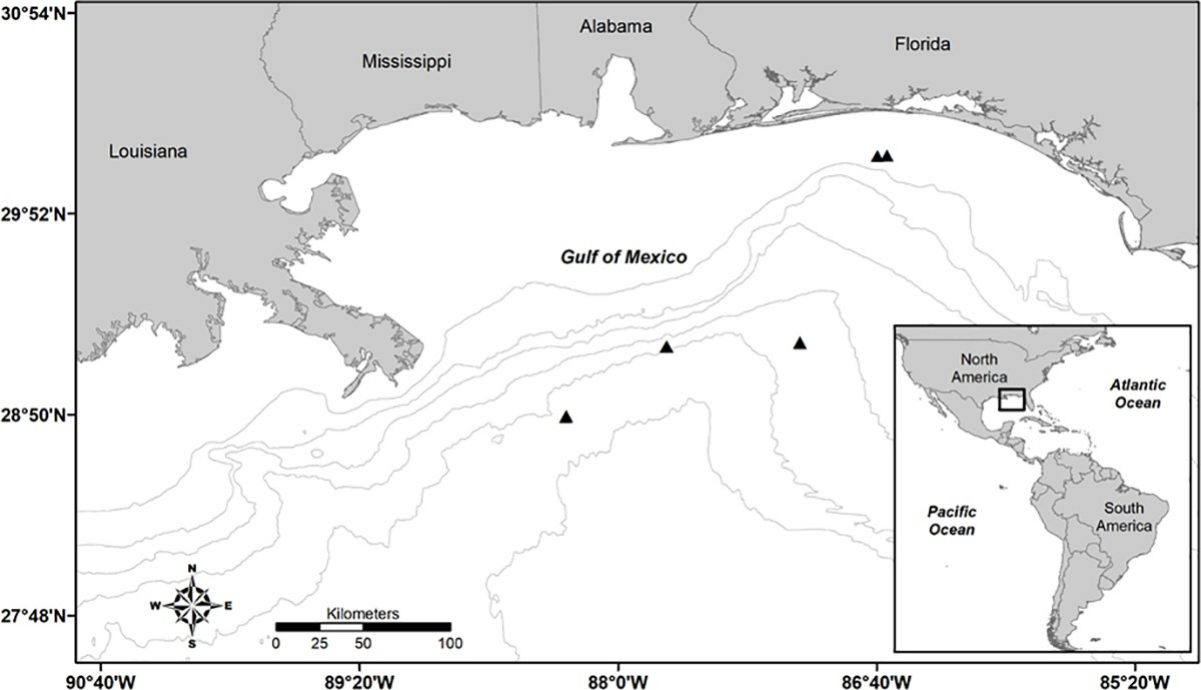
Northern Gulf of Mexico map showing water sampling locations. Map of DeSoto Canyon sampling locations in the northern Gulf of Mexico (black triangles) where water samples were taken for analysis of dissolved inorganic carbon Δ^14^C. The 50, 100, 200, 500, 1000 and 2000 m isobaths are shown. The thicker line is the 200 m isobath, which is the depth where the outer continental shelf ends and the slope waters begin. This map was made using ArcGIS software.

The distribution of dissolved inorganic carbon (DIC) Δ^14^C with depth was investigated by collecting seawater in tripped bottles at depths of 5–1,248 m from stations within the DeSoto Canyon located in the nGOM (Fig 2). Water was recovered on deck and filtered with combusted GFF Whatman glass-fibre filters, and then stored in pre-evacuated glass vials sealed with butyl stoppers. One mL of 20% H_3_PO_4_ was added to each sample to preserve for shipment to Florida State University. Samples were prepared by He stripping and cryogenic trapping into 6 mm Pyrex tubes and sent to NOSAMS for Δ^14^C analysis. A linear regression was fit to the DIC Δ^14^C values from DeSoto Canyon stations located in 200–600 m depths since these profiles are more likely to be associated with outer shelf and upper slope areas where Warsaw grouper would be found [1,33].

An attempt was made to fit a von Bertalanffy growth function (VBGF) to Warsaw grouper length-age data with the method of least squares, but results were not biologically plausible given the parameter estimates produced from the VBGF function (e.g., t_0_ = -9.02 y; k = 0.043 y^-1^; and L_∞_ estimated to be 2,034 mm). Therefore, we utilized the method of Taylor et al. [34] to fit the VBGF, which estimates and accounts for gear selectivity, M, and fishing mortality (F) within a Bayesian framework. The Taylor et al. [34] approach assumes logistic selectivity, that size-at-age data are sampled from a multinomial distribution, and that recruitment variation, M, and F have been stable for a number of years so as to provide a stable size-age distribution for the population. Uninformative (weak) priors were placed on the VBGF parameters, the CV on length, TL mm at 50% selectivity, and the steepness of the logistic selectivity curve (Table 1). M was initially estimated based on the method of Hewitt and Hoenig [35] and maximum observed longevity as 0.069 y^-1^ (see below). This value served as the prior on M in the Taylor et al. [34] model, which was treated as an informative prior (Table 1). Prior values for VBGF parameters were based on the Manooch [36] parameter estimates for Warsaw grouper collected from the Atlantic Ocean waters off the southeastern U.S. (Table 1). Models also were computed with alternative priors on L_∞_ (with the same standard deviation as shown in Table 1) of 1,687 and 1,850 mm TL, which are the mean length of fish estimated to be ≥20 y and >30 y old, respectively, in the observed nGOM data used in this study. The prior on the length at 50% selectivity (880 mm) is the mean length of fish <15 y old in the observed data, with a weak prior on the slope of the selectivity function indicating a gradual slope.

**Table 1:** Log-normal prior and posterior means and standard deviations for von Bertalanffy growth function, logistic selectivity, and instantaneous natural and fishing mortality variables. The model handles the variables in log space; standard deviation of each parameter is given in log space.

	Prior		Posterior	
Parameter	Value	SD	Value	SD
L_∞_ mm TL	1,618	5	1,533	0.03
k y^-1^	0.13	2	0.14	0.07
t_0_ y^-1^	0.2	2	1.82	0.09
CV on length	0.12	2	0.17	0.02
TL mm at 50% selectivity	880	5	812	0.02
logistic curve steepness	5	2	78.8	0.06
M y^-1^	0.069	0.1	0.066	0.10
F y^-1^	0.2	2	0.34	0.04

The population length-age composition was simulated with the Taylor et al. [34] model given observed length-age data and priors described above, and then VBGF parameters were fit to the simulated data. Posterior distributions were estimated for model parameters using the Metropolis-Hastings method MCMCmetrop1R implemented in the R package “MCMCpack” [37] in the program R [31]. Four chains were used for each parameter estimation, with a burn in of 5,000 and a sample of 200,000. Chain convergence was visually tested and was confirmed with the Gelman and Rubin multiple sequence diagnostic, Geweke diagnostic, and Heidelberg and Welch convergence diagnostic from the R package “coda” [38].

## Results

Northern GOM Warsaw grouper samples ranged in TL from 133 to 2,186 mm, and age estimates ranged from 1 to 61 y (n = 1,338). A subsample (36%; n = 485) of sectioned otoliths was aged by a second reader. The APE between the two readers’ age estimates was 10.0% with a CV of 14.1%, which is higher than the generally accepted APE precision reference point of <5.5% (CV of 7.6%) for moderately long-lived species and moderate reading complexity [10]. An overall percent reader agreement for the 485 samples was 51%. Age estimates between reader one and reader two were within ±1 year for 79% of the samples and within ±2 years for 91% of the samples.

**Fig 3:**
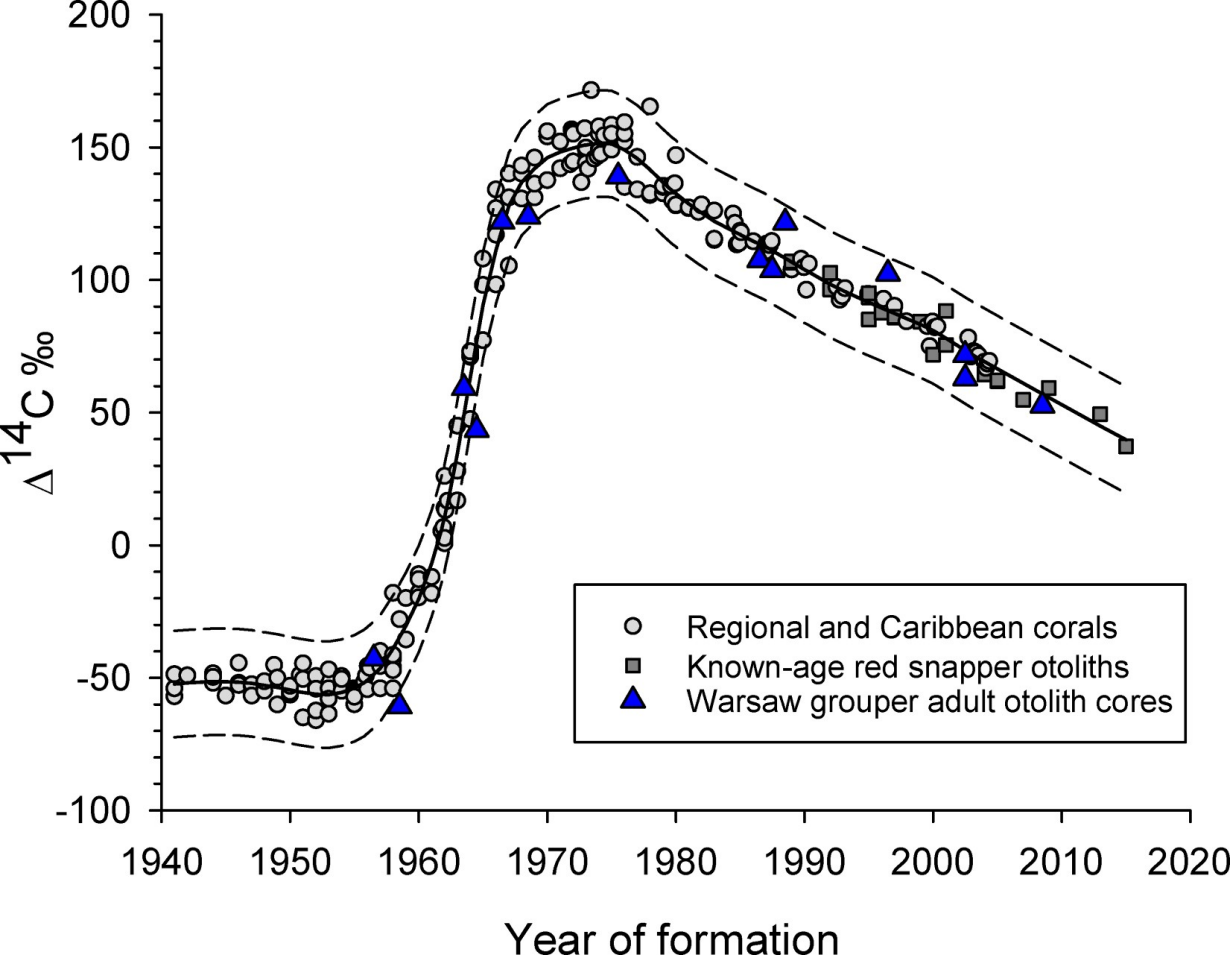
Regional coral and known-age otolith Δ^14^C values versus year of formation with Warsaw grouper otolith core Δ^14^C values overlain on plot. Scatterplot of regional coral and known-age red snapper *Lutjanus campechanus* otolith Δ^14^C values versus year of formation, with Warsaw grouper otolith core Δ^14^C data overlain on the plot. A loess regression (degree = 2; α = 0.20) fit to the coral and known-age red snapper otolith Δ^14^C values is shown as a solid line. Dashed lines are Bonferroni-corrected 95% confidence intervals. Warsaw grouper birth year was estimated as sample collection year minus otolith opaque zone counts.

**Fig 4:**
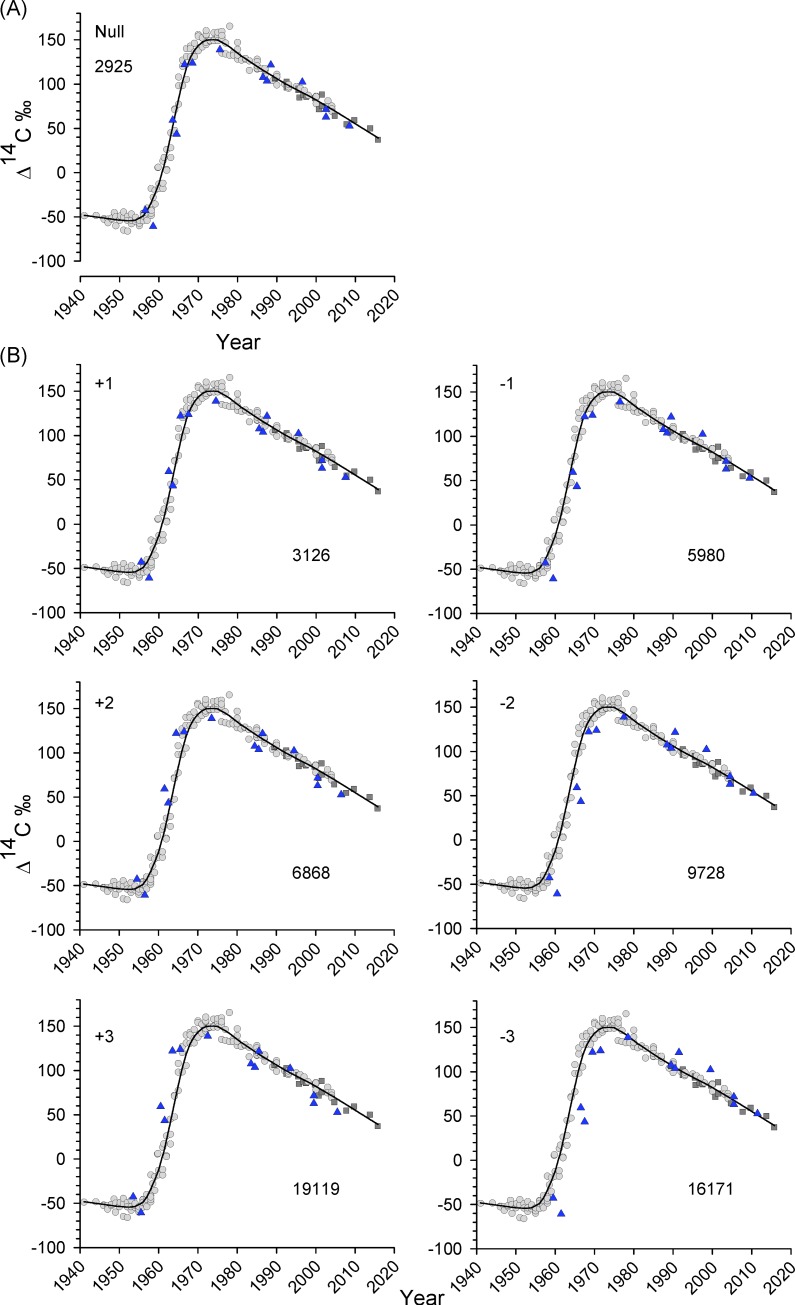
Bias plots of Warsaw grouper otolith core Δ^14^C values. Bias plots of Warsaw grouper otolith core Δ^14^C values relative to the -loess regression fit to regional coral data (Fig 3). Panel A shows original age estimates, which had the lowest SSR. Panel B plots are labeled with purposely shifted ages (±1 to ±3 years). The sum of squared residuals (SSR) is shown on each panel and calculated following the method of Kastelle et al. [32].

Age estimates ranged from 1 to 59 y for Warsaw grouper samples (n = 14) whose otolith cores were extracted and analyzed for Δ^14^C with AMS (Table 2). Estimated birth year (year of collection minus age) corresponded well with the coral-otolith Δ^14^C time series (Fig 3). The maximum directly validated age for Warsaw grouper was 54 y (birth year = 1963; Table 2; Fig 3). A sample with estimated age of 59 y (Fig 1) could not be validated given its birth year (1958) occurred just prior to the start of the rise period; however, its Δ^14^C is nonetheless consistent with an estimated birth year of 1958. Bias plots of Warsaw grouper otolith core Δ^14^C values produced SSRs for the age-shifted estimates that ranged from 3,126 (+1 year) to 19,119 (+3 years), while the SSR for the original age estimates was 2,925 (Fig 4).

**Table 2:** Warsaw grouper otolith Δ^14^C samples. Warsaw grouper otolith samples analyzed for Δ^14^C with AMS. Otoliths for which no otolith mass or age estimate was recorded are shown as—. Year of formation equals sample year minus estimated age. NM = not measured.

Sample number	Analysis number	TL mm	Otolith mass mg	Sample date	Year of formation	Age years	δ^13^C ‰	Δ^14^C ‰
WRG-1	OS-93156	1158	0.389	08/21/1980	1966	14	−2.84	122.06±5.0
WRG-2	OS-93154	942	0.307	04/22/1991	1986	5	−4.92	107.59±4.7
WRG-3	OS-93155	1007	0.350	06/05/1991	1987	4	−3.61	103.77±4.7
WRG-4	OS-93085	1007	0.416	01/07/1992	1988	4	−4.05	121.73±5.1
WRG-5	OS-93300	1760	1.557	06/10/2000	1975	25	−4.15	139.05±5.3
WRG-6	OS-93157	1442	0.862	09/05/2004	1996	8	−2.71	102.42±4.8
WRG-7	OS-94285	1206	0.500	10/18/2009	2002	5	−3.24	62.98±3.1
WRG-0022	OS-132877	203	0.021	12/22/2005	2008	1	−4.46	52.73±2.2
WRG-0403	OS-133006	2134	1.979	04/22/2005	1956	49	−1.73	−42.7±1.9
WRG-1465	OS-133007	1805	1.591	12/12/2013	1964	41	−1.94	43.51±2.1
WRG-3201	OS-133008	1994	--	12/12/2013	1968	45	−1.91	123.94±2.3
WRG-3203	OS-133009	1403	--	12/12/2013	2002	11	−3.78	71.74±2.1
WRG-0675	OS-138429	1954	1.956	02/09/2017	1958	59	NM	−60.72±2.1
WRG-17-SPO	OS-138430	2186	2.447	08/19/2017	1963	54	−2.97	59.29±2.2

**Fig 5:**
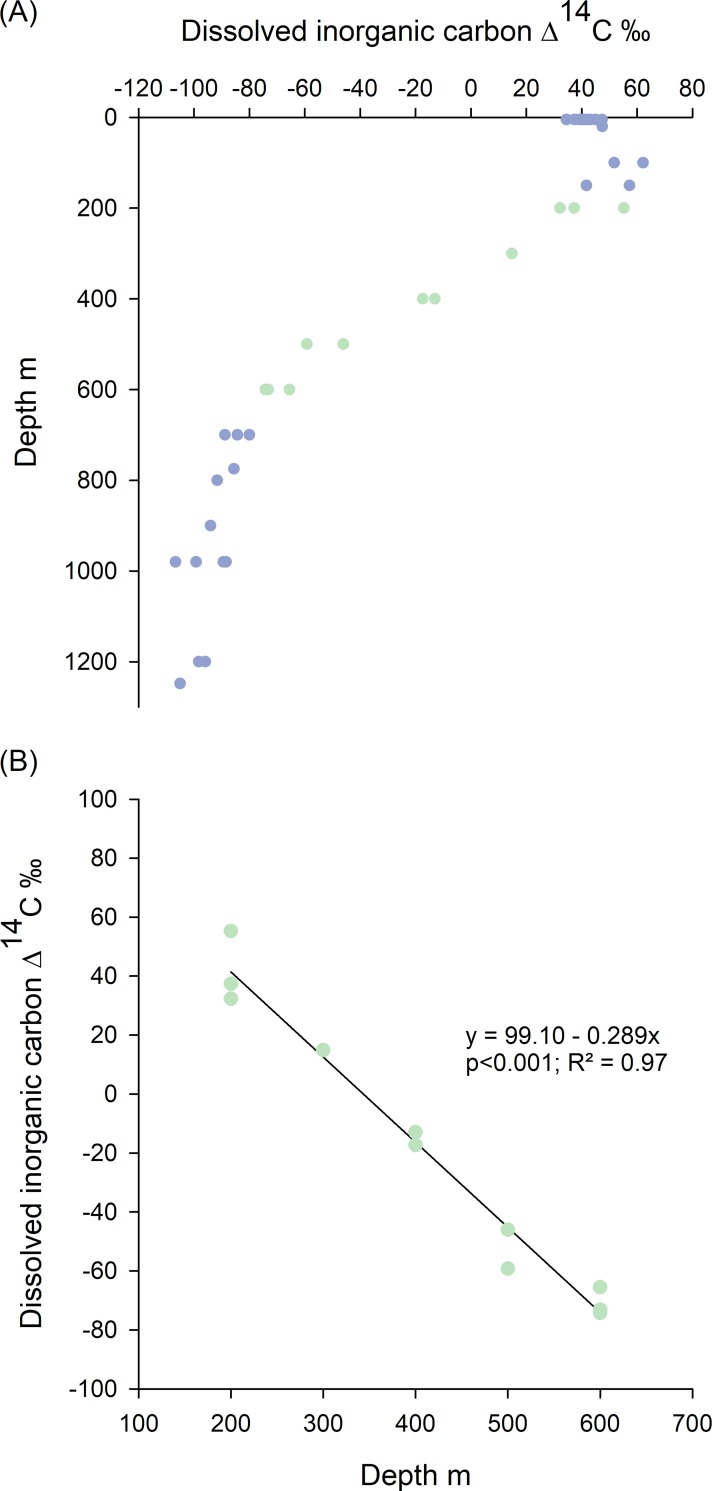
Northern Gulf of Mexico DIC Δ^14^C values sampled at DeSoto Canyon. Panel A is a profile of nGOM DIC Δ^14^C sampled at DeSoto Canyon stations (Fig 2) by depth. Green data points represent 200–600 m depth, which is the depth range where Warsaw grouper have been reported to reside. Panel B is a linear regression fit to the DIC Δ^14^C values for the 200–600 m depth range.

Seawater DIC, which was used to draw inference about likely juvenile Warsaw grouper habitat, had bomb-produced Δ^14^C values in subsurface waters to approximately 200 m depth (Fig 5A). Depths >200 m had DIC depleted in ^14^C with depth (Fig 5A). A linear regression fit to the DIC Δ^14^C values from DeSoto Canyon stations within the 200–600 m depth range, which is the depth range most likely associated with outer shelf and upper slope areas where Warsaw grouper are likely to be found [1,33], showed a significant linear relationship (p < 0.001; R^2^ = 0.97, n = 11) with a slope of -0.289 ‰ per m (Fig 5B).

**Fig 6:**
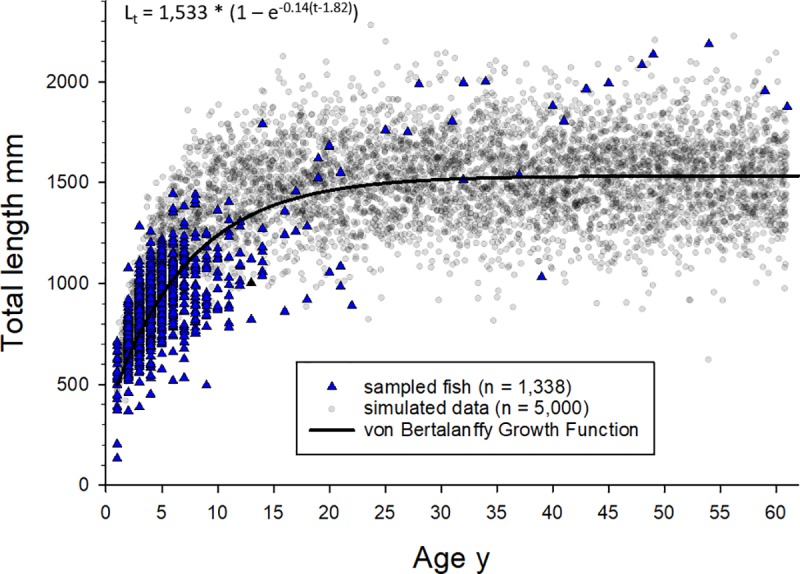
Von Bertalanffy growth function fit to observed and simulated Warsaw grouper length-age data. Von Bertalanffy growth function fit to observed (blue triangles; n = 1,338) and simulated (gray circles; n = 5,000) nGOM Warsaw grouper length-age data utilizing the Bayesian model described by Taylor et al. [34]; model priors and estimated parameters are listed in Table 1.

Estimated VBGF parameter (posterior) values were L_∞_ = 1,533 mm, k = 0.14 y^-1^, and t_0_ = 1.82 y (Table 1, Fig 6). M was estimated to be 0.066 y^-1^ and F was estimated to be 0.34 y^-1^. Therefore, Z (F + M) was estimated as 0.406 y^-1^ and the estimated F:M was 5.1:1. Models fit with the prior on L_∞_ set to 1,687 or 1,850 mm produced the exact same estimates for all Taylor et al. [34] model parameters as the fit with the L_∞_ prior set to 1,618 mm (Table 1).

## Discussion

Study results indicate counts of nGOM Warsaw grouper otolith opaque zones provide accurate age estimates as validated based on application of the bomb radiocarbon chronometer, with the oldest single age directly validated being 54 y. Two additional fish examined in this study had estimated ages of 59 and 61 y based on otolith opaque zone counts. Therefore, while 54 y was the maximum age for a single otolith sample directly validated with Δ^14^C, the validated age estimation process produced age estimates to 61 y. Based on this maximum age observation and utilizing the approach of Hewitt and Hoenig [35], M was estimated as 0.069 y^-1^. Application of the Taylor et al. [34] model to Warsaw grouper length-age data, while utilizing the Hewitt and Hoenig [35] estimate as a prior, produced an estimate for M of 0.066 y^-1^. Both of these values are consistent with other long-lived reef fishes in the region [39–41].

The fact that Warsaw grouper otolith core Δ^14^C values were relatively indistinguishable from shallow water coral Δ^14^C values indicates juvenile Warsaw grouper likely spend at least their first 6 months of life in shelf waters <200 m. Waters within this depth range are well-mixed with respect to anthropogenic ^14^C input from atmospheric nuclear bomb testing that occurred during the 1950s – 1960s, which has subsequently mixed out of surface layers and into the deeper ocean. Using Δ^14^C from fish otoliths to estimate life-history stages of fish that are rarely encountered was applied to smooth oreo *Pseudocyttus maculatus*, with results suggesting juvenile smooth oreo live in surface waters [42]. Results of more recent bomb radiocarbon studies also successfully linked the juvenile life-history stage of deepwater fishes to the upper-ocean mixed layer (*Hyperoglyphe Antarctica*, [43]; *Epigonus telescopus*, [44]).

The DIC Δ^14^C values collected from the nGOM further support the inference that Warsaw grouper juveniles occur in shelf waters <200 m. DIC Δ^14^C values in this depth range, which are similar to the Δ^14^C values obtained from Warsaw grouper otolith cores, are enriched with bomb-produced ^14^C relative to DIC Δ^14^C values in waters >200 m. Likewise, since ~70–90% of otolith carbon is derived from seawater DIC [45], it could be expected that otolith Δ^14^C would be similar to DIC Δ^14^C values [18]. The Δ^14^C values collected within the DeSoto Canyon from depths of 200–600 m, which is the depth range in which Warsaw grouper are generally thought to reside, show a change in Δ^14^C values of approximately -29‰ for every 100 m increase in depth. Therefore, the similarity of seawater DIC Δ^14^C values at depths <200 m and Warsaw grouper otolith core Δ^14^C values support the inference that juvenile Warsaw grouper reside in shelf waters.

This study is the first age and growth study conducted for Warsaw grouper in the nGOM. Manooch and Mason [46] reported a maximum age of 41 y for Warsaw grouper from the Atlantic Ocean waters off the southeastern U.S. In the current study, the maximum age for the species from the nGOM had an estimated observed age of 61 y and a maximum directly validated age of 54 y. For many deepwater teleosts, opaque zones in otoliths are often difficult to interpret due to surrounding environmental constancy of bathypelagic, abyssal and bathyal environments. This can result in aging error, which consists of two components: bias and imprecision [10−11,47]. An age validation process is performed to assess the accuracy of age estimates derived from growth zone counts in aging structures, such as otolith opaque zones, and APE is a statistically valid way to measure aging precision [10,48]. Thus, Δ^14^C results in this study validate the accuracy of Warsaw grouper age estimates, and calculated APE provides a measurement of precision between otolith readers. Although the APE of 10.0% reported in this study is relatively high given the generally accepted APE of <5.5% [10], it is not unexpected given that authors of other studies on deepwater species have reported relatively high APEs. For example, an APE of 10.4% was reported for goldband snapper *Pristipomoides multidens* [49] and an APE of 11.9% (CV of 16.8%) was reported for yellowedge grouper *Hyporthodus flavolimbatus* from the nGOM [50]. Furthermore, it has been questioned whether an APE of 5.5% should be utilized as a threshold for species with otolith sections that are difficult to interpret [51]. Although nGOM Warsaw grouper otolith thin sections can be difficult to age, results from the current study provide support that accurate and precise age estimates can be derived for this species. Accuracy of Warsaw grouper age estimates was validated using bomb radiocarbon Δ^14^C, and there was no indication that age estimates were biased given results of applying the Kastelle et al. [32] method to Warsaw grouper birth year estimates and otolith core Δ^14^C values.

Growth modeling initially produced mixed results. The VBGF follows an assumption that length data are representative for each age class; however, this assumption cannot be met since sample collection is often size-selective and size-at-age can be affected by the cumulative effects of fishing [34]. This can lead to length-age data being biased since size-selective harvesting will remove fast-growing individuals while slower growing individuals elude capture [52]. There is also evidence, not only in the current study, but also for other deepwater teleosts that length and age can be highly variable and can become decoupled so that length of a fish is not indicative of the age [4,49].

The relatively recent likelihood method described in Taylor et al. [34] was utilized in the current study to estimate the von Bertalanffy growth parameters while accounting for the effects of gear selectivity along with M and F across the age range of individuals. The shift in L_∞_ below its initial prior may suggest that without a prior a lower estimate of L_∞_ would result. However, the prior on L_∞_ was relativity weak (uninformative) and the same posterior estimate of L_∞_ (1,533 mm TL) resulted regardless of whether higher or lower prior values were stipulated. In addition, the lack of a posterior update on instantaneous M indicates that this parameter is not estimable from the current data and only total mortality can be estimated; therefore, any estimate of F is conditioned on the prior used for M. That said, the estimate of M was based on an established life-history linked method [35], and it is unlikely that the actual M was substantially higher than 0.069 y^-1^ given the validated longevity observed for this species. Furthermore, while a least squares fit of the VBGF to the length-age data was achieved, parameter estimates were not biologically plausible given the severely right-truncated age distribution, where 94% of the ages are <10 y (refer to Fig 6). The application of Taylor et al.’s [34] Bayesian model allowed us to simulate the length composition of the population, given observed length-age composition and priors on selectivity and M, and then to estimate VBGF parameters and F. The model was insensitive to different values of the prior on L_∞_, as it converged on the same parameter estimates for each of the L_∞_ prior values utilized.

The simulated length-age data produced with the Taylor et al. [34] model have a wide distribution of size-at-age for the age classes >20 y, which was informed by length-age observations for younger fish, M, and estimated selectivity. The exact same model results were produced when the prior on L_∞_ was changed to 1,687 or 1,850 mm TL, but that was not unexpected given the prior was uninformative (weak), thus the model fit was not constrained near any of the values input as priors on L_∞_. The sparse data for Warsaw samples >20 y old encompassed the range in the simulated data of approximately L_∞_ ± 500 mm, or ± 33% of L_∞_. Such a range is not uncommon, and several nGOM marine fishes have ranges in size-at-age for older age classes of a similar or greater magnitude relative to estimated L_∞_ [53–55], including other grouper species [56–57]. Further, the Brody growth rate coefficient for Warsaw grouper from the nGOM was higher than that reported for Warsaw grouper from the Atlantic Ocean off the southeastern U.S., k = 0.14 y^-1^ versus k = 0.054 y^-1^ [46]. It is also possible that growth may differ between males and females, such as reported for another protogynous hermaphrodite, yellowedge grouper *Hyporthodus flavolimbatus*, from the nGOM where VBGF predicted females to grow faster and reach a smaller asymptotic length than males [41]. However, since there were insufficient sex data available for the current study, it was not possible to test for potential differences between sexes.

Warsaw grouper in the nGOM, like many deepwater groupers around the globe, are vulnerable to exploitation because they are long-lived, display slow growth, mature late in life, and have low M [1–3,58]. While no formal stock assessment has been conducted to date for nGOM Warsaw grouper, estimates of F (0.34 y^1^) and F:M (5.1:1) were produced in the current study. As a general rule, overfishing occurs when F exceeds M [59], and Zhou et al. [60] estimated that on average for teleosts F at maximum sustainable yield equals 0.87M. While uncertainty in the absolute estimate of F (0.34 y^-1^) exists for nGOM Warsaw grouper because of the sparseness of data and assumptions for applying the Taylor et al. [34] method, an F:M estimate of 5.1:1 is consistent with the inference that Warsaw grouper biomass is severely depleted throughout its range [21–22]. Although the F or F:M estimates reported here may not suffice as even a data-poor stock assessment of nGOM Warsaw, they serve as the first estimates of fishing mortality for this stock, and they corroborate qualitative estimates that nGOM Warsaw grouper historically have undergone substantial fishing effort and their biomass is likely to be significantly depleted in the region. Estimates we report provide further impetus to conduct a formal stock assessment for nGOM Warsaw grouper.
