# Correction: Life history of northern Gulf of Mexico Warsaw grouper *Hyporthodus nigritus* inferred from otolith radiocarbon analysis

**DOI:** 10.1371/journal.pone.0245403

**Published:** 2021-01-07

**Authors:** The PLOS ONE Staff

There is information missing from the Funding statement. The complete, correct Funding statement is as follows: This research was made possible in part by a grant from NOAA Fisheries, Office of Protected Resources, Species of Concern Program, in part by a grant from The Gulf of Mexico Research Initiative, and in part by funding from the Florida Fish and Wildlife Conservation Commission (Grants FWC-08304 and FWC-16188 to WFP). Riverside Technology, Inc. provided support in the form of salary for author LT, but did not have any role in the study design, data collection and analysis, decision to publish, or preparation of the manuscript. The specific roles of this author are articulated in the 'author contributions' section.

The publisher apologizes for the error.

The following information is missing from the Data Availability statement: Dissolved inorganic carbon 14C data are publicly available through the Gulf of Mexico Research Initiative Information & Data Cooperative (GRIIDC) at https://data.gulfresearchinitiative.org (doi:10.7266/N7W37TWR).
